# The rehabilitation nutrition oral care process: Implementing the triad of rehabilitation, nutrition, and oral management

**DOI:** 10.1002/jgf2.763

**Published:** 2025-02-06

**Authors:** Hidetaka Wakabayashi

**Affiliations:** ^1^ Department of Rehabilitation Medicine Tokyo Women's Medical University Hospital Tokyo Japan

## Abstract

A specific framework called the rehabilitation nutrition oral care process has been developed to facilitate the triad of rehabilitation, nutrition and oral management. Each framework follows five key steps: assessment, diagnosis, goal setting, intervention, and monitoring. Of these, the diagnosis and goal setting steps are performed collaboratively by multidisciplinary teams specializing in rehabilitation, nutrition and oral management.

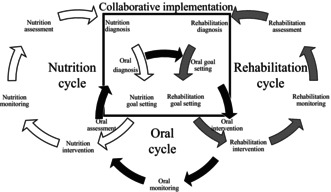

The 2024 revisions to Japan's medical and long‐term care reimbursement systems emphasize the coordination and promotion of rehabilitation, nutrition and oral management. These revisions aim to address the challenges of Japan's super‐aging society by preventing decline in function and quality of life (QOL) due to conditions such as hospital‐associated deconditioning and iatrogenic sarcopenia during hospitalization, and maintaining or improving these functions and QOL after discharge. Evidence has shown that a triad of rehabilitation, nutrition and oral management can lead to improvements in swallowing function, activities of daily living, weight gain in individuals with low body mass index, and increases in muscle mass and strength.^1^, ^2^, ^3^ A specific framework called the rehabilitation nutrition oral care process has been developed to facilitate the implementation of this coordination.

The rehabilitation nutrition oral care process is an enhanced version of the simplified rehabilitation nutrition care process.^4^ While the simplified rehabilitation nutrition care process focuses on one cycle of rehabilitation and nutrition, the new framework includes three cycles‐rehabilitation, nutrition and oral management. Each framework follows five key steps: assessment, diagnosis, goal setting, intervention, and monitoring. Of these, the diagnosis and goal setting steps are performed collaboratively by multidisciplinary teams specializing in rehabilitation, nutrition and oral management.

Rehabilitation diagnosis is used to determine the presence and causes of sarcopenia and to identify disability and its causes using the International Classification of Functioning, Disability and Health. Nutritional diagnosis focuses on the identification of undernutrition, overnutrition, and imbalances in nutrient intake, including their underlying causes. Oral diagnosis focuses on oral functional decline, oral hygiene problems, and oral environmental problems and their causes. These diagnoses may be performed by physical therapists, occupational therapists, and speech‐language pathologists for rehabilitation; registered dietitians for nutrition; and dentists and dental hygienists for oral management. However, the sharing of diagnostic findings among interdisciplinary teams is critical to ensuring the triad of rehabilitation, nutrition and oral management (Figure 1).

**FIGURE 1:**
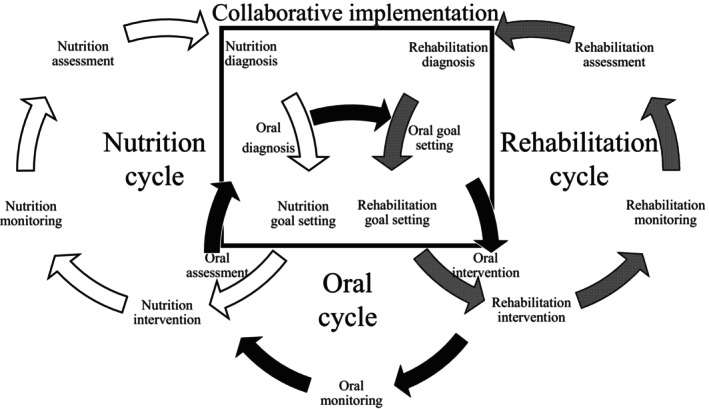
Rehabilitation nutrition oral care process. The rehabilitation nutrition oral care process consists of three cycles: The rehabilitation cycle, the nutrition cycle and the oral cycle. All cycles have five steps: Assessment, diagnosis, goal setting, intervention and monitoring. Diagnosis and goal setting for rehabilitation, nutrition and oral are done collaboratively. Physical therapists, occupational therapists and speech‐language pathologists carry out the rehabilitation cycle. Registered dietitians carry out the nutritional cycle. Dentists and dental hygienists carry out the oral cycle. All cycles should take place at the same time. Doctors and nurses coordinate the rehabilitation nutrition oral care process.

Goal setting for rehabilitation, nutrition, and oral management should adhere to the SMART framework, which ensures that goals are Specific, Measurable, Achievable, Relevant, and Time‐Bound (SMART).^5^ General goals such as “improve nutrition” or “improve oral health” do not meet the SMART criteria. Instead, examples of SMART goals include improving oral conditions to a Revised Oral Assessment Guide (ROAG) score of 8 within 2 weeks, fabricating dentures that provide bilateral occlusal support in all four posterior regions within 1 month, or achieving independence in consuming texture‐modified diets and weaning from tube feeding within 1 month. Because nutritional and oral goals can influence rehabilitation goals, it is important to establish these goals together.

Doctors and nurses are encouraged to serve as coordinators of the rehabilitation, nutrition and oral care process. While they do not need to have in‐depth expertise in all three areas, they should be able to identify issues related to rehabilitation, nutrition and oral management in the clinical setting. Their responsibilities include prescribing rehabilitation, requesting nutrition counseling or assistance from nutrition support teams, and consulting with dental and oral management teams. In addition, they should facilitate interdisciplinary conferences to share diagnostic findings and collaboratively set goals.

If the triad of rehabilitation, nutrition, and oral management is not yet feasible, it is practical to begin by promoting collaboration between any two of these domains—for example, rehabilitation and nutrition, rehabilitation and oral care, or nutrition and oral care. This initial two‐domain collaboration should include bi‐directional perspectives, such as understanding nutrition from a rehabilitation perspective or vice versa. Once this two‐domain collaboration is achieved, efforts can then move toward the triad of rehabilitation, nutrition, and oral care. Application of the rehabilitation‐nutrition‐oral care process in primary care settings is expected to contribute to improved function and QOL.

## CONFLICT OF INTEREST STATEMENT

Hidetaka Wakabayashi is an Editorial Board member of the Journal of General and Family Medicine and an author of this article. To minimize bias, he was excluded from all editorial decision‐making related to the acceptance of this article for publication. The author declares no conflict of interest for this article.

## References

[1] Wakabayashi H , Kakehi S , Kishima M , Itoda M , Nishioka S , Momosaki R . Impact of registered dietitian and dental hygienist involvement on functional outcomes in patients with dysphagia: triad of rehabilitation, nutrition, and oral management. Eur Geriatr Med. 2023;14(6):1301–1306. 10.1007/s41999-023-00833-7 37442874

[2] Wakabayashi H . Triad of rehabilitation, nutrition, and oral management for sarcopenic dysphagia in older people. Geriatr Gerontol Int. 2024;24(Suppl 1):397–399. 10.1111/ggi.14651 37577770

[3] Yoshimura Y , Shimazu S , Shiraishi A , Wakabayashi H , Nagano F , Matsumoto A , et al. Triad of rehabilitation, nutrition support, and oral management improves activities of daily living and muscle health in hospitalized patients after stroke. Clin Nutr ESPEN. 2024;63:837–844. 10.1016/j.clnesp.2024.08.018 39181533

[4] Kakehi S , Isono E , Wakabayashi H , Shioya M , Ninomiya J , Aoyama Y , et al. Sarcopenic dysphagia and simplified rehabilitation nutrition care process: an update. Ann Rehabil Med. 2023;47(5):337–347. 10.5535/arm.23101 37907225 PMC10620494

[5] Wakabayashi H , Mori T , Nishioka S , Maeda K , Yoshimura Y , Iida Y , et al. Psychological aspects of rehabilitation nutrition: a position paper by the Japanese Association of Rehabilitation Nutrition (secondary publication). J Gen Fam Med. 2023;25(1):1–9. 10.1002/jgf2.668 38240004 PMC10792333

